# We’ve Come a Long Way, Baby: Announcing a Special Issue to Commemorate the Publication of Molecule’s 20,000th Paper

**DOI:** 10.3390/molecules25010173

**Published:** 2019-12-31

**Authors:** Farid Chemat, Roman Dembinski, Arnaud Gautier, James W. Gauld, Derek McPhee, Diego Muñoz-Torrero, Joselito P. Quirino, Thomas J. Schmidt, Vadim A. Soloshonok, Mark von Itzstein

**Affiliations:** 1 *Molecules* Green Chemistry Section Editor-in-Chief; 2 *Molecules* Organic Chemistry Section Editor-in-Chief; 3 *Molecules* Organometallic Chemistry Section Editor-in-Chief; 4 *Molecules* Theoretical Chemistry Section Editor-in-Chief; 5 *Molecules* Editor-in-Chief; 6 *Molecules* Medicinal Chemistry Section Editor-in-Chief; 7 *Molecules* Analytical Chemistry Section Editor-in-Chief; 8 *Molecules* Natural Products Chemistry Section Editor-in-Chief; 9 *Molecules* Organic Chemistry Section Editor-in-Chief; 10 *Molecules* Bioorganic Chemistry Section Editor-in-Chief

*We’ve Come a Long Way, Baby*
Title song of the 1979 album of the same name by American country-music composer and singer Loretta Lynn

On behalf of my Section Editor-in-Chief co-author colleagues I am pleased to announce a Special Issue to commemorate the recent publication of *Molecules’* 20,000th paper. For this we invite the submission of high quality reviews and articles on any of the subjects covered in *Molecules’* eighteen topical sections.

While a country-western song title may seem out of place in a *Molecules* Editorial, the words nevertheless seem most appropriate to define the journal’s trajectory since volume 1 was published in 1996, to the occasion we now aim to celebrate with the Special Issue being announced herein. Between Chattopadhyay et al.’s Synthesis of the Demospongic Compounds, (6*Z*,11*Z*)-Octa-decadienoic Acid and (6*Z*,11*Z*)-Eicosadienoic Acid [1], the first non-editorial paper published in what was then one of the first e-journals in the field, to the paper we now celebrate, Cagide et al.’s Optimizing the Synthetic Route of Chromone-2-carboxylic Acids: A Step forward to Speed-up the Discovery of Chromone-based Multitarget-directed Ligands [2], *Molecules* and its publisher MDPI AG have indeed come a very long way in a very short time [3] and much has happened in these 23 plus years, during which our growth has been nothing short of spectacular, as witnessed by Figure 1, which shows the years some of our milestone papers were published. 

*Molecules* is now one of the preeminent Open Access journals in the field of chemistry, with a 2018 Journal Citation Report^®^ Impact Factor of 3.060 and a 5-Year Impact Factor of 3.380 (2018) [4]. It ranks 68/172 (Q2) in the category “Chemistry, Multidisciplinary” and 136/298 (Q2) in “Biochemistry and Molecular Biology.” MDPI AG’s trajectory over these years has been even more spectacular [5], but perhaps only one number will suffice to illustrate this: *Molecules* now has 213 sibling journals. 

As for the mechanics of this Special Issue, we anticipate a 90-day submission period, following the normal submission process, during which the Section Editors and I will be selecting the manuscripts that move on to standard anonymous peer review. To encourage publication of high quality papers, and submissions on underrepresented topics or from underserved regions, each Section Editor will have access to a certain number of page charge waivers, to be used at their discretion. In summary, we would like to invite all chemists and scientists working in related areas to submit their best work to this Special Issue, as well as to *Molecules*. 

## Figures and Tables

**Figure 1:**
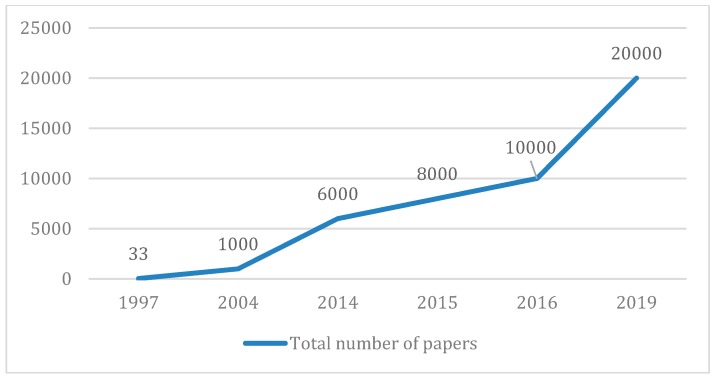
The years some milestone papers were published.
